# Smoking Cessation Counselling for Pregnant and Postpartum Women among Midwives, Gynaecologists and Paediatricians in Germany

**DOI:** 10.3390/ijerph6010096

**Published:** 2009-01-05

**Authors:** Kathrin Röske, Wolfgang Hannöver, Jochen René Thyrian, Ulrich John, Hans-Joachim Hannich

**Affiliations:** 1 Ernst-Moritz-Arndt-University Greifswald, Institute for Medical Psychology, Walther-Rathenau-Str. 48, 17487 Greifswald, Germany; E-mails: hannoeve@uni-greifswald.de (W. H.); hannich@uni-greifswald.de (H. -J. H.); 2 Ernst-Moritz-Arndt-University Greifswald, Institute for Community Medicine, Department of Community Health, Ellern-Holz-Str. 1–2, 17487 Greifswald, Germany; E-mail: thyrian@uni-greifswald.de; 3 Ernst-Moritz-Arndt-University Greifswald, Institute of Epidemiology and Social Medicine, Walther-Rathenau-Str. 48, 17487 Greifswald, Germany; E-mails: ujohn@uni-greifswald.de

**Keywords:** Smoking cessation counselling pregnancy

## Abstract

The incorporation of guidelines for the treatment of tobacco smoking into routine care requires positive attitudes, counselling skills and knowledge about additional help available for smokers. The study assesses performance of smoking cessation intervention, attitudes, training status and knowledge about additional help for smokers in the care for pregnant and parenting women by midwives, gynaecologists and paediatricians. A survey of all midwives, gynaecologists and paediatricians registered for primary medical care in the federal state Saarland, Germany, was conducted. Participation in the postal questionnaires was 85 %. Depending on profession, 90 % to 100 % see smoking cessation counselling as their assignment, 17 % to 80 % screen for, 48 % to 90 % document smoking status, and 55 % to 76 % offer brief or extensive counselling. 61 % to 87 % consider training to enhance their knowledge and/or counselling skills necessary. The compliance of providers with the necessity to give support in smoking cessation is very high. However, the current status of cessation counselling does not sufficiently correspond to the evidence based requirements. Reports in medical press and advanced training courses should support health care providers and establish smoking as an inherent topic of the anamnesis and treatment of current and former pregnant or parenting smokers.

## 1. Introduction

Smoking during pregnancy remains the most important preventable risk factor for fetal death, low birth weight and other complications of pregnancy [1]. The exposure of infants to environmental tobacco smoke (ETS) increases the risks for respiratory diseases, otitis media and sudden infant death syndrome [2] and is responsible for hospitalisation [3]. In Germany 35 % to 47 % of women smoke before pregnancy, while estimates about quitting rates during pregnancy range from 49 % to 56 % [4, 5]. About half of the women who quit during pregnancy resume smoking within one year after delivery [6].

A growing volume of research has demonstrated that there are effective interventions to achieve abstinence from smoking [7]. For the special population of pregnant and parenting smokers specific programmes for smoking cessation and relapse prevention were tested. Metaanalyses and reviews conclude that interventions reveal small to moderate effects [8, 9]. Effectiveness is enhanced with growing intensity of the intervention and by using pregnancy-specific written materials [10, 11].

The evidence led to the development of guidelines for the treatment of tobacco smoking demanding that every patient using tobacco should be offered at least a brief intervention [7, 12]. During pregnancy, treatment should exceed minimal advice, and include pregnancy-specific self-help-materials. Intervention should be offered at the first visit as well as at all subsequent visits throughout the course of pregnancy and delivery [7, 13]. These guidelines address all health professionals. In Germany they were published by the task force of the scientific medical associations [14]. Little is known whether they are implemented in the treatment of pregnant and postpartum women. This should be part of tobacco control policy. However, Germany is known to be among the least active countries in Europe in this area [15, 16].

Opportunities to address smoking during and after pregnancy are obviously present. In Germany, gynaecologists, midwives and paediatricians routinely provide health care for these women. Due to the continuous contact to the women these health professionals are in an excellent position to address tobacco use. First, during pregnancy there are monthly gynaecological check-ups up to the 32nd week of gestation and further check-ups every two weeks up to delivery. In the postpartum period gynaecological well women visits provide continued opportunity to approach the topic. Second, every woman is entitled to utilise services provided by midwives. These comprise twelve counselling contacts and monthly check ups during pregnancy, participation in antenatal courses, daily home visits within 10 days after delivery and six more contacts up to eight weeks after delivery. Third, there are six routine checkups for the newborn within the first year after delivery. Usually from the third checkup (between weeks four and six after delivery), these take place in paediatric practices.

Further prerequisites for the implementation of guidelines obviously seem to be individual factors of those who provide counselling, such as: positive attitudes towards smoking cessation counselling, counselling skills as well as knowledge about pregnancy specific self-help materials. In Germany, little is known about these individual prerequisites. Up to now professional groups were surveyed separately at different points in time which impedes comparison between groups. A survey of all paediatricians in the Federal State of Mecklenburg-West-Pomerania in the northeast of Germany (participation rate 62 %) revealed that 26 % screened for smoking status, 47 % of them documented smoking status in the patient’s record and offer smoking cessation counselling according to their self-statements in a questionnaire [17]. A survey of all midwives in the same study region (participation proportion 77 %) revealed that a considerably higher proportion of the respondents, 77 % screen for smoking and offer brief counselling [18].

The objective of this study was to describe and compare a) the screening and smoking cessation counselling behaviour and b) attitudes, training status and knowledge about additional materials for smokers of professional groups most intensively dealing with pregnant or postpartum women in a country with low activities in smoking prevention such as Germany.

## 2. Methods

### 2.1. Survey

The study region Saarland is a federal state in the southwest of Germany, and is part of the former West Germany. The presence of 1.05 million inhabitants in the smallest federal state by land area results in a higher-than-average population density (411 residents per km^2^; average for all Germany: 231 inhabitants per km^2^). Outpatient medical care is provided on a fee for service basis by physicians of different specialties, like gynaecologists and paediatricians. The costs are paid by the patient’s health insurance. Every patient is free to consult her physician of choice. The same rules apply for utilization of services from midwives.

At the time of the survey, between May and August 2006, 130 gynaecologists and 72 paediatricians were registered for primary medical care in the study region. According to the health statistics of Saarland there are about 170 midwives working in the study region [19]. Since midwives are not registered by name in these statistics and there is no complete register of midwives, a search for contact data was conducted for midwives working in hospitals of Saarland or having their own independent practices in this federal state. Different sources were used: the register of members of the Federation of German Midwives (regional federation of Saarland), regional public health departments, phone books and world wide web. The search revealed contact data for 246 midwives. Thus, contact data of 448 professionals were available.

In January 2006, 204 providers (128 midwives, 50 gynaecologists, 26 paediatricians) were randomly selected and invited to participate in a trial aimed at the implementation and evaluation of a training course in smoking cessation counselling skills. Providers received a letter including a questionnaire and a pre-paid return envelope. They were asked to complete this survey irrespective of their readiness to participate in the evaluation study. In May 2006 all remaining 244 providers (118 midwives, 80 gynaecologists, 46 paediatricians) in the study region (not contacted for trial recruitment) received a letter asking for completion of the survey. For these providers two items addressing the wish to participate in advanced training about pre- and postpartum smoking and possible interventions were added to the original questionnaire.

When questionnaires were not returned within four weeks providers were reminded by phone or by mail if they could not be reached by phone. No incentives were used for recruitment. Persons not wanting to participate could indicate this at any time on the phone, on the questionnaire or by not answering at all.

### 2.2. Questionnaire

The smoking counselling attitudes and practice questionnaire was based on well accepted versions used in earlier surveys [17, 18], therefore we abstained from a pretest. It consisted of four main topics:

*Screening and counselling behaviour*: Participants were asked for screening and repeatedly addressing smoking behaviour of their patients on a 5-point Likert scale (from 1 “always” to 5 “never”), respectively. Answers were dichotomised into screening “always” vs. screening “not always”. This reflects adherence to the requirements of the guidelines. Additional information pertaining to this topic were documentation of smoking status and of changes in smoking behaviour in the patients’ record (yes/no), and the extent of counselling: “no counselling”, “advice only”, “brief counselling (less than 10 minutes)”, and “counselling > 10 minutes”. A question on brochures and self-help materials aimed at whether these are passively displayed at the practice/office or are actively handed out to the patients.

*Attitudes towards smoking cessation counselling during and after pregnancy*: the importance of counselling of currently smoking women and of women who stopped smoking in pregnancy was asked on 10-point-rating-scales ranging from 1 “not important” to 10 “very important”. The perceived certainty in counselling was measured from 1 “not certain at all” to 10 “very certain” and the estimated chance of success of such counselling was measured on a scale from 1 “very low” to 10 “very high”. Regarding the question whose assignment it should be to counsel pregnant and postpartum women participants could mark one or more of the following professions: gynaecologists, paediatricians, midwives and nursing staff.

*Training status*: one question addressed whether professionals had ever attended specialised training on smoking cessation counselling (yes/no). If so, they were asked to describe this training in an open question. As described above, the questionnaire for the providers contacted in the second part of the survey (May 2006) included two additional items. First, it was asked whether the health care provider would like to participate in an advanced theoretical training giving information about smoking in the context of pregnancy and treatment (yes/no) and if so, of which extent it should be. Second, it was asked whether they wished to participate in a practical or smoking cessation counselling skills training (yes/no) and if so which extent this training should have. The extent of theoretical and practical trainings was dichotomized into either written material only (e.g. newsletter, brochures, articles, counselling manuals) or workshops (brief, one-day-, weekend-workshop, more extensive).

*Characteristics of the respondent*: data on age, sex, professional years and smoking status of the respondents were collected.

### 2.3. Sample

**Figure 1.**
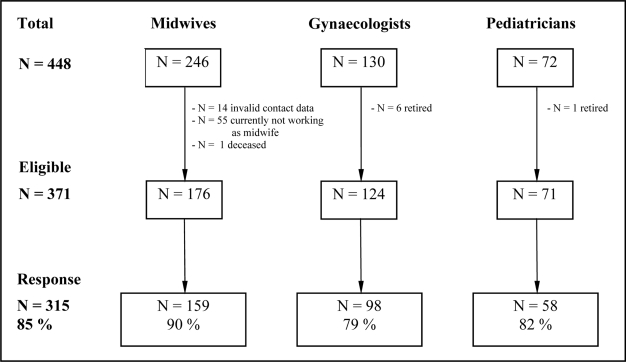
Recruitment of the sample in the federal state Saarland, Germany, by profession. Sample characteristics are displayed in Table 1.

Of the 130 gynaecologists, six had retired by the time of the survey, leaving 124 gynaecologists eligible. Of the 72 paediatricians one had retired, leaving 71 paediatricians eligible. Within the contact data of 246 midwives 14 were revealed to be invalid and no correct data could be obtained, 55 women were currently not working as midwifes (e.g. retirement, maternity leave, had not finished their vocational education yet), and one had deceased, leaving 176 eligible midwives, which corresponds to the earlier mentioned health statistics. The resulting sample size for the survey consisted of 371 health care providers of which 85 % completed the survey (Figure 1).

### 2.4. Statistical Analysis

Descriptive statistics were calculated using SPSS 12.0. The reported percentages refer to valid data. Calculation of 95%-confidence intervals (95%-CI) for percentages and means was performed according to Altman *et al*. [20].

## 3. Results

### 3.1. Screening and Counselling Behaviour

While three quarter of the midwives and gynaecologists reported to routinely assess the smoking status of every woman during the first contact, only 17 % of the paediatricians did so. About 90 % of the midwives and gynaecologists documented smoking status in the patient’s record compared to half of the paediatricians (Table 2). The proportion of addressing smoking in every subsequent contact varied between 23 % among the gynaecologists and 5 % among the paediatricians. More changes in smoking status were documented by midwives and gynaecologists than by paediatricians (29%).

A minority of 10 % of the health care providers reported that they offer more than 10 minutes counselling. In contrast, up to 40 % of the practitioners gave advice only. The vast majority of the respondents did not have self-help brochures or did not use them.

### 3.2. Attitudes towards Smoking Cessation Counselling during and after Pregnancy

The importance of smoking cessation counselling was rated very high in all professional groups, with medians of 9 and 10 for the responses on a 10-point rating scale (Table 3). In contrast, the importance of counselling women who already attained abstinence was generally lower (medians from 5 to 6).

The respondents reported a high certainty in counselling women (medians 7 to 8). The chance of success of smoking cessation or relapse prevention counselling was rated lower by all professionals (medians 3 to 5), whereas paediatricians where the most pessimistic.

The vast majority of respondents saw smoking cessation and relapse prevention counselling as their task, as well as task of the two other professional groups of interest. About 40 % found that counselling about smoking should be the duty of nursing staff, too.

### 3.3. Training

Eleven persons, 4 % of all 315 respondents, reported ever having participated in specialised training on smoking cessation counselling (midwives: 4, gynaecologists: 4, paediatricians: 3). When analysing the open answers on the kind of training two persons gave no description, five did not name tobacco smoking-specific trainings (e.g. training in acupuncture or hypnosis), four had participated in a tobacco smoking-specific training.

Within the sub-sample of 187 persons who were asked whether they had personal interest in an advanced training in the field of tobacco smoking 74 % perceived a need for either a theoretical or a counselling skills training. These were 87 % of the midwives, 61 % of the gynaecologists and 67 % of the paediatricians. In detail, the wish for theoretical training on smoking in the context of pregnancy was reported more often compared to wishes for practical skills training by all professionals (table 4). When asked for the extent of such theoretical training 79 % of midwives, 74 % of the gynaecologists and 76 % of the paediatricians considered workshops necessary while a minority of providers preferred written material only. When asked for the required extent of a practical counselling skills training 76 % of midwives, 77 % of the gynaecologists and 83 % of the paediatricians preferred workshops, while a minority wanted written material only, such as counselling manuals.

## 4. Discussion and Conclusions

Our data reflect discrepancies between the apparent need for prevention and evidence based guidelines on the one hand, which partly express in positive attitudes of professionals towards smoking cessation counselling, and self-reported implementation on the other hand. Almost all providers consider cessation counselling a very important task of their specific profession. Furthermore, they perceive the same responsibility for the respective other professions. However, systematic screening is not well implemented. The extent of counselling is usually very brief or advice only. Although most midwives and gynaecologists document smoking status in the patient’s record, this information is not routinely updated and also not consequently used for addressing smoking in every subsequent contact. Only a minority of the professionals is provided with self-help brochures. Especially paediatricians differ in reporting their screening and counselling activities compared to their claim. Compared to the expressed high importance of smoking cessation counselling the relevance of relapse prevention is rated significantly lower. Given the high relapse rates which are reported nationally [6] and internationally [21–23] there is still too little attention spent on women who quit spontaneously. Thus, current practice misses opportunities to support women in abstaining from smoking. In particular relapse prevention and prevention of environmental tobacco smoke for the baby is disregarded.

Two reasons for this might be derived from the survey: first, the perceived low chances of success of smoking cessation counselling and second, the lack of tobacco specific specialised training. It seems plausible that the reported strong motivation of midwives and physicians to participate in advanced trainings is based on these aspects. It reflects the hope to learn new and more effective treatment approaches. This motivation is an enormous potential for optimizing care for mothers and babies by training. The fact that almost nobody among the respondents had participated in a tobacco specific training reflects the lack of offers for training in Germany rather than a lack of interest.

We assume representativeness of our results for Germany for two reasons. The very high response rate is a strength of our study compared to earlier surveys reporting response rates of 62 % to 77 % in samples of discrete professional groups [4, 17, 18]. However, in a study on self-reported socially desired behaviour nonresponders might be those who are not interested in prevention of tobacco smoking of their patients. Therefore some selection bias cannot be excluded. Second, our results correspond to the findings of these earlier surveys conducted in other regions of Germany. What our study adds is a direct comparison of those three professional groups dealing with pregnant and parenting women on an updated comprehensive database.

Comparisons of our results with international data seem problematic because of largely differing health services and differences in the national anti-tobacco climate. Nevertheless, some apparent similarities according to implementation of smoking prevention in the context of pregnancy and childbearing could be mentioned. Studies from the USA and New Zealand on smoking cessation counselling behaviour of different professional groups reported consistently that the vast majority asks their patients about smoking, most give advice to stop but only few report to go beyond those steps by offering counselling or assistance in stopping smoking, arranging follow-up contacts on smoking etc. [24–28]. Thereby paediatricians showed the lowest smoking intervention practice [29]. Furthermore, participants of these surveys frequently expressed their pessimism about effectiveness of counselling and mentioned a lack of training, those important barriers for counselling revealed by our study, too.

The results of our survey underline the necessity for more support of health care providers in prevention activities in order to forward implementation of guidelines. Specialised training about smoking and treatment is wanted by professionals and must be offered to teach effective counselling skills. In this process two simple tools that could improve implementation should be communicated: every provider should establish an early detection scheme by screening for current *and* former smoking behaviour. Consistent documentation of smoking status should serve as reminder for the necessity of smoking cessation counselling in every subsequent contact as well as relapse prevention in the woman who is abstinent for months. Respective data might be noted in the patients record and in the record of prenatal and natal care every expectant mother receives from her gynaecologist after detection of pregnancy and which she should carry with her for all subsequent pre- and the first postnatal contacts to her gynaecologist and midwife.

Additional information must be provided about available written materials, such as self-help brochures for patients, self-instruction guides for providers and about further treatment options. Respective materials, such as a counselling manual for gynaecologists, a manual for paediatricians and numerous brochures and leaflets, are already available for free from different institutions in Germany, including the Federal Center for Health Education. As our data reflect materials at hand are rarely accompanied by or used as a prompt for personal counselling. However, a study of Lang *et al*.[4] revealed that the proactive distribution of self-help booklets at regular time intervals to gynaecologists and paediatricians enhanced the use of such materials and increased counselling.

Overall the results of the present study point to the individual learning needs among all professional groups dealing with pregnant and parenting women. They must be supported by respective training targeting providers screening activities, knowledge about successful counselling strategies, use of supporting materials and their confidence in being effective and by a growing anti-tobacco climate on the policy-makers side. Surveys like the one presented here are capable for monitoring the development of tobacco specific health care for pregnant and postpartum women. It might be the initial point for future evaluation after implementation of urgently necessary activities like the development of educational programs. Furthermore the survey is the basis for observation of possible changes in counselling behaviour during the next years since further tobacco control activities and attitude changes can hopefully be expected in Germany. On an international scale there have been efforts to monitor tobacco control activities in a more complex way [30]. Our survey could contribute to the emerging evidence in Germany.

## Figures and Tables

**Table 1.** Sample characteristics.

	Midwives n = 159	Gynaecologists n = 98	Paediatricians n = 58
**Age (M, SD)**	37.4 (9.2)	51.3 (7.7)	48.8 (7.8)
**Years in profession (M, SD)**	13.9 (9.5)	23.3 (8.1)	22.1 (8.1)
**% Female**	100	41	40
**% Current smoker**	20	9	10

*Notes*: M = arithmetic mean; SD = standard deviation.

**Table 2.** Smoking cessation counselling behaviour of midwives, gynaecologists and paediatricians in Saarland, Germany.

	Total (n = 315) % (95%-CI)	Midwives (n = 159) % (95%-CI)	Gynaecologists (*n*= 98) % (95%-CI)	Paediatricians (*n* = 58) % (95%-CI)
***At the primary contact***				
Routinely (always) screening for smoking status	67 (61.1–71.6)	76 (69.2–82.4)	80 (71.1–86.9)	17 (9.6–28.9)
Documenting smoking status in medical record	81 (76.4–85.0)	90 (84.1–93.6)	87 (78.4–92.0)	48 (35.9–60.8)
***Subsequent contacts***				
Routinely (always) addressing smoking	15 (11.6–19.5)	14 (9.4–20.3)	23 (15.6–32.2)	5 (1.8–14.1)
Documenting changes in smoking behaviour	59 (53.4–64.3)	58 (50,1–65.4)	78 (69.2–85.4)	29 (19.2–42.0)
***Extent of counselling***				
Not at all	2 (1.1–4.6)	3 (1.0–6.4)		5 (1.8–14.1)
Advice only	26 (21.5–31.2)	21 (15.5–28.2)	26 (18.1–35.3)	40 (28.1–52.5)
Brief counselling (< 10 min.)	61 (55.9–66.7)	65 (57.6–72.4)	61 (50.9–69.9)	52 (39.2–64.1)
Counselling >10 minutes	10 (7.4–14.2)	11 (6.9–16.8)	13 (8.0–21.6)	3 (1.0–11.7)
***Use of self-help-brochures***				
No brochures at hand	74 (68.8–78.5)	83 (76.0–87.8)	59 (48.8–68.0)	76 (63.5–85.0)
Brochures are displayed	19 (14.7–23.4)	10 (6.4–16.0)	32 (23.5–41.8)	19 (10.9–30.9)
Brochures are handed over	7 (5.0–10.9)	7 (4.0–12.2)	9 (5.0–16.7)	5 (1.8–14.1)

**Table 3.** Attitudes of midwives, gynaecologists and paediatricians towards smoking cessation counselling.

	Total (n = 315)	Midwives (n = 159)	Gynaecologists (*n* = 98)	Paediatricians (*n* = 58)
	**M**	**M**	**M**	**M**
***Attitudes towards counselling***	**(SD; 95%-CI)**	**(SD; 95%-CI)**	**(SD; 95%-CI)**	**(SD; 95%-CI)**
Importance of counselling smoking pregnant women or women post partum 1	8.9 (1.7; 8.7–9.1)	8.9 (1.6; 8.7–9.2)	9.4 (1.2; 9.2–9.6)	8.1 (2.3; 7.5–8.7)
Importance of counselling women who stopped smoking during pregnancy 1	6.2 (2.8; 5.9–6.5)	6.4 (2.7; 6.0–6.8)	6.2 (2.8; 5.6–6.8)	5.7 (2.8; 4.9–6.5)
Perceived certainty in counselling about smoking 1	7.3 (2.3; 7.0–7.6)	6.9 (2.4; 6.5–7.3)	8.2 (1.8; 7.8–8.6)	6.8 (2.2; 6.2–7.4)
Estimated chance of success of counselling 2	4.3 (1.9; 4.1–4.5)	4.3 (1.9; 4.0–4.6)	4.9 (1.9; 4.5–5.3)	3.1 (1.5; 2.7–3.5)
Counselling about smoking should be the task of	**% (95%-CI)**	**% (95%-CI)**	**% (95%-CI)**	**% (95%-CI)**
Gynaecologists	97 (94.1–98.2)	97 (92.7–98.6)	100 (96.2–100)	91 (80.7–96.1)
Paediatricians	86 (82.1–89.8)	89 (83.2–93.1)	80 (71.4–87.1)	89 (78.5–95.0)
Midwives	89 (84.7–91.7)	99 (95.4–99.6)	77 (68.0–84.5)	80 (68.2–88.7)
Nursing staff	41 (35.1–46.0)	44 (36.1–51.4)	33 (24.4–42.8)	45 (32.4–57.6)

*Notes*: M = arithmetic mean; SD = standard deviation;

1 continuous variable, scale ranged from 1 “not important/certain” to 10 “very important/certain”,

2 continuous variable, 1 “very low” to 10 “very high”.

**Table 4.** Wish for advanced training on the topic of smoking cessation within a sub-sample of midwives, gynaecologists and paediatricians.

	Total (n = 315) % (95%-CI)	Midwives (n = 159) % (95%-CI)	Gynaecologists (*n* = 98) % (95%-CI)	Paediatricians (*n* = 58) % (95%-CI)
Wish for theoretical training/information about smoking	70 (62.9–76.1)	83 (73.4–89.5)	57 (44.1–68.1)	64 (48.4–77.3)
Wish for skills training on smoking cessation counselling	54 (46.3–60.6)	61 (50.2–70.8)	48 (36.4–60.6)	46 (31.6–61.4)
